# Wound Healing with Electrical Stimulation Technologies: A Review

**DOI:** 10.3390/polym13213790

**Published:** 2021-11-01

**Authors:** Yt Jun Cheah, Muhamad Ramdzan Buyong, Mohd Heikal Mohd Yunus

**Affiliations:** 1 Department of Physiology, Universiti Kebangsaan Malaysia Medical Centre, Kuala Lumpur 56600, Malaysia; cheahytjun@yahoo.com; 2 Institute of Microengineering and Nanoelectronics, Universiti Kebangsaan Malaysia, Bangi 43600, Selangor, Malaysia; muhdramdzan@ukm.edu.my

**Keywords:** electrical stimulation, skin, tissue engineering, wound healing

## Abstract

Electrical stimulation (ES) is an attractive field among clinicians in the topic of wound healing, which is common yet complicated and requires multidisciplinary approaches. The conventional dressing and skin graft showed no promise on complete wound closure. These urge the need for the exploration of electrical stimulation to supplement current wound care management. This review aims to provide an overview of electrical stimulation in wound healing. The mechanism of galvanotaxis related to wound repair will be reviewed at the cellular and molecular levels. Meanwhile, different modalities of externally applied electricity mimicking a physiologic electric field will be discussed and compared in vitro, in vivo, and clinically. With the emerging of tissue engineering and regenerative medicine, the integration of electroconductive biomaterials into modern miniaturised dressing is of interest and has become possible with the advancing understanding of smart biomaterials.

## 1. Introduction

Chronic wounds are commonly encountered in patients with comorbidities [1] and not only physically, mentally, and financially impact the patient but also jeopardise the economy of a country [2]. In the UK’s National Health Service, an estimated £8.3 billion was spent on managing an estimated 3.8 million wound cases in 2017/2018 with a 71% increase in the prevalence from 2012/2013 [3]. Meanwhile, Medicare in the United States estimated a cost of $32 billion in wound care in 2014 [4]. In Germany, the prevalence of chronic wounds in 2012 was estimated to be 1.04% [5]. Hence, chronic wounds impose a profound economic burden, and the wound care market is predicted to reach $15–22 billion by 2024 [1]. 

Despite the increasing varieties of wound dressing materials available in the market, chronic wounds do not respond fast to conventional dressings. Although it is the gold standard treatment of chronic wounds, skin grafting is short of standardisation of the harvested graft thickness. It also possesses a high risk of graft failure, donor site infection, and pain over both surgical sites [6,7]. Meanwhile, skin substitutes such as Dermagraft, Apligraft, and Affinity are high cost and lack evidence [8]. It is crucial to reassess the pathology underlying a chronic wound when recovery is slower than expected. In the literature, ES is one of the recommended advanced wound-care modalities [9,10].

ES is an exogenous application of the electrical field to accelerate the process of wound healing by mimicking the natural current of an injury [11,12,13]. Directed cell migration under the influence of a naturally occurring electric field is known as galvanotaxis. This cue overrides other co-existing factors such as chemotaxis, wound void, population pressure, mechanical forces, injury stimulation, and contact inhibition release in the context of epithelial wound healing [14]. In view that chronic wounds have a weaker wound current that slows down cell migration, proliferation, and differentiation [15], ES is expected to restore the current of injury and expedite wound recovery.

Posifect and Procellera are among the marketed ES. Posifect is a battery-driven device with two-electrodes placement, while Procellera is a wireless patch driven by the electrochemical reaction between zinc and silver [16]. Procellera is approved by the FDA solely as an antimicrobial wound dressing. However, there is no ES device or dressing approved by FDA to promote wound healing. 

ES for wound-care purposes is still in clinical trials and not widely practised, although potential benefits have been proven. Therefore, ES shall be highly highlighted in a wound-related context and aims to replace painful surgical-based skin grafts in the future. In this paper, skin battery and current of injury in wounded skin in addition to the mechanism of the wound-healing process related to ES are introduced. Different modalities of ES such as direct current, pulsed current, and alternating current are discussed together with in vitro, in vivo, and clinical studies. Lastly, new technologies of ES such as electroconductive materials, nanogenerator, and bioelectric plaster-like wound dressing are reviewed. This review paper aims to provide an insight into the role of ES and the aspect of translation into clinical application in wound care management.

### 1.1. Skin as an Endogenous Battery

**Figure 1:**
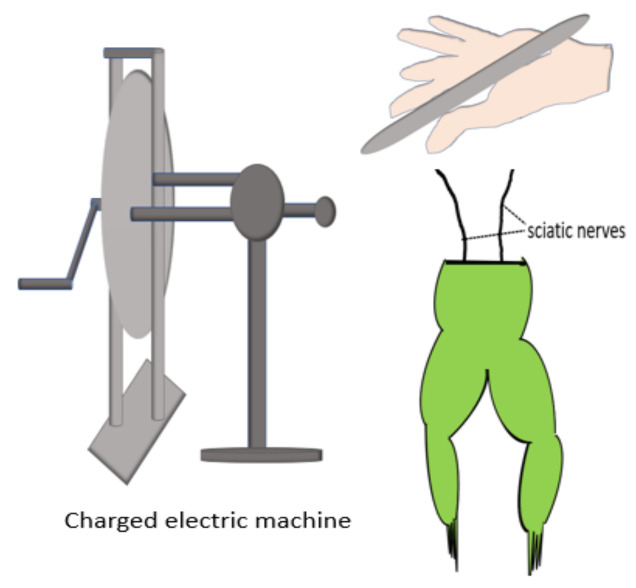
Luigi Galvani and the prepared frog next to the spark experiment. A prepared frog consisted of both the lower limbs with the internal crural nerves exposed from the spinal cord level, and a metal wire was inserted across the vertebral canal and positioned a distance away from a charged electrical machine. The frog’s legs contracted powerfully when an assistant of Galvani, most likely his wife Lucia Galeazzi Galvani, accidentally touched the internal crucal nerves with a lancet. In the meantime, a spark was observed from the nearby electrical machine. This observation led Galvani to study animal electricity.

**Figure 2:**
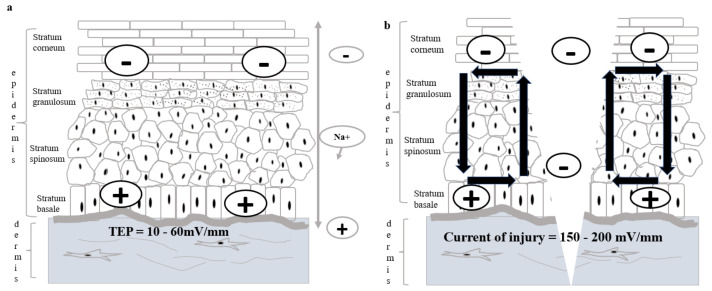
TEP of a normal skin is generated by Na/K/ATPase pump where the Na^+^ ions are continuously pumped into skin. The TEP is measured to be 10-60 mV/mm in which the anucleated stratum corneum is relatively negative compared to the stratum basale as shown in subfigure (**a**). Subfigure (**b**) shows the current of the injury when the skin is wounded. As the TEP is disturbed during an injury, the center of a wound experiences a drop in potential, making it more negatively-charged comssspared to the wound edges. The potential difference between the edges and center of a wound generates the current of injury of 150–200 mV/mm which directs the most skin cells to migrate to the center of the wound in the healing process and closes up the wound.

In 1781, Luigi Galvani (1737–1798) found that a frog’s legs contracted when the exposed internal crural nerves were touched by a scalpel. Galvani stated that it was “animal electricity” as shown in Figure 1. [17,18,19]; however, a study by Alessandro Volta (1745–1827) concluded that electricity was produced by the contact between different metals, and the frog only acts as a passive conductor [20]. In 1843, DuBois-Reymond recognised the values in both Galvani’s and Volta’s experiments and constructed a galvanometer, which detected the current of injury of approximately 1 µA for the first time [21]. Their works led to the current understanding of electrophysiology, electromagnetism, electrochemistry, and electrical battery [20]. The human epidermis is found to be relatively negatively-charged compared to the dermis, and there is a negligible potential difference between any part of the dermis. This is known as skin battery or transepithelial potential (TEP) ranging between 10 and 60 mV/mm in the human epidermis as shown in Figure 2a, with the hairless skin having a higher reading compared to the hairy skin [22,23]. TEP is generated by the asymmetric transport of charged ions in the epidermis by Na/K/ATPase pumps [24,25], and the main ions involved are sodium, potassium, calcium, and chloride [26]. 

### 1.2. Role of an Endogenous Electric Field in Wound Repair

When the epithelium is wounded, TEP will be disrupted, and the electric field will be short-circuited as shown in Figure 2b. The centre of the wound is found to have a drop in potential compared to the surrounding [22,27]. This potential difference creates an endogenous lateral electrical field, which directs the current of injury from the wound edge towards the wound centre [26,28]. This trend of current of injury is also observed in another study on rat and human skin wounds [27]. The endogenous current of the injury in the wounded human epidermis was measured to be in the range of 150–200 mV/mm, and the value reaches zero when wound healing was completed [22,29,30,31]. In a rat skin model, approximately 3 µAcm^−2^ of the current of injury was measured, and the current was sustained for hours [32]. 

## 2. ES Approaches in Promoting Wound Healing

**Figure 3:**
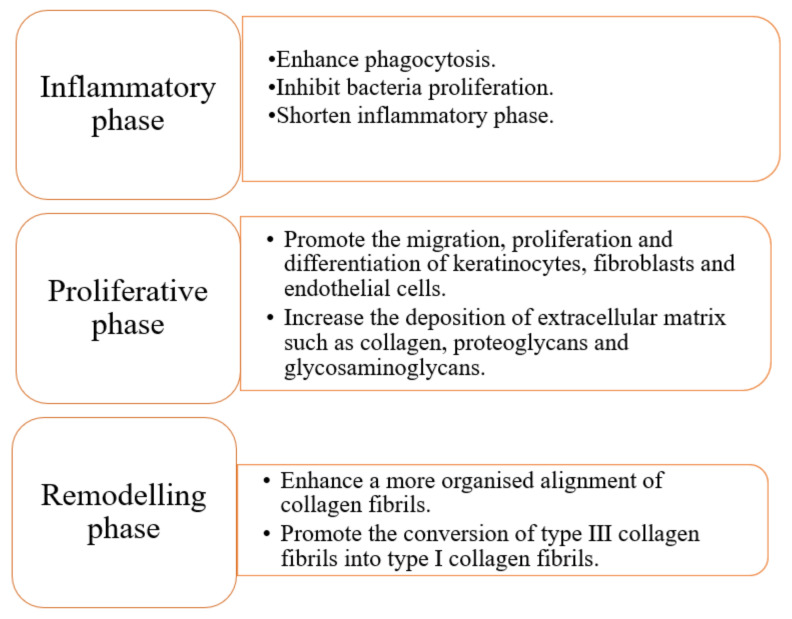
The effects of ES on the major outcomes of wound healing.

As the largest organ in humans, the skin serves mainly as a barrier protecting humans from external environmental insults [33]. A wound is formed when there is a break in the continuity of the skin affecting its physiological function. Disruption of the normal TEP generates a current of injury. This paper will review the mechanism of current of injury in the sophisticated wound-healing process involving distinctive yet overlapping phases of inflammation, proliferation, and remodelling [34,35]. Figure 3 shows the overall effects of ES on wound healing.

### 2.1. Inflammatory Phase 

The inflammatory phase occurs immediately after a wound is formed and includes a coagulation cascade, inflammatory pathway, and activation of the immune system [36]. ES promotes vasodilation and increases vascular permeability so that more cells including leukocytes and platelets can be recruited to the wounded area [37,38]. Hoare et al. (2016) showed that macrophages migrated to the anode at a very sensitive level of 5 mV/mm, and the speed of the directed migration was proportional to the field strength. Macrophages exposed to physiological electric field strength (150 mV/mm for 2 h) are parallelly aligned to the applied external field vector with a polarised redistribution of polymerised actin, podosomes, and phagocytic receptors. This leads to an enhanced phagocytosis of carboxylate microspheres, C albicans, and apoptotic neutrophils. At the molecular level, the electric field activates ERK and P13K pathways that subsequently increase intracellular calcium influx such as TRPV2 -like calcium influx [39] in macrophages to enhance bacteria phagocytosis efficiency [40]. 

Regarding bacteria issues in inflammation phase, there have been extensive studies as early as the 19th century. DC of milliampere (0.2–140 mA) inhibited the growth of E. coli, while AC of similar intensity (current 15–30 mA) had a negligible outcome [41]. In another study, the lower intensity (0.4–400 microampere) of DC inhibited Staphylococcus aureus [42]. In recent years, there are only a few studies on bacteria using ES. A Bacteriostatic effect was found on Staphylococcus epidermidis through the application of AC [43]. A study on *E. coli* found that DC caused two-way leakage in the membrane that is large enough to make way for protein leaking, thus killing the bacteria [44]. Another study using AC (5 and 20 mA) inhibited growth of Pseudomonas aeruginosa, but no effect was seen on Escherichia coli and Staphylococcus aureus [45]. 

### 2.2. Proliferative Phase 

The proliferative phase of wound healing involves re-epithelisation, fibroplasia, and angiogenesis. ES encourages keratinocyte proliferation and differentiation with more deposition of keratin in addition to an elevated migration speed of keratinocytes, which is a prerequisite for re-epithelisation [46,47]. Another study demonstrated a similar finding in which electric field-exposed keratinocytes were directed to the cathode with increased directedness that is proportional to the electric field strength, while control without ES showed random migration [48]. Meanwhile, the ERK1/2 and p38 MAP kinase pathways are the upregulated pathways during the ES on keratinocytes, which is associated with the reduction of proinflammatory cytokines IL-6 and IL-8 [46]. The downregulation of proinflammatory cytokine expression suggest the effective transition from the inflammatory phase to the proliferative phase [49]. An ex vivo study on the human wound with both monophasic and biphasic ES of the field strength of 100 mV/mm for 30 min daily for 16 days accelerated the granulation tissue ingrowth into the wound centre. A significantly greater staining of cytokeratin-10 in the immunohistochemical analysis and approximately three times higher cytokeratin-10 mRNA expression compared to control at day 16 with double the epidermal thickness was observed [50]. In this ex vivo wound model, ES was found to significantly upregulate PCNA, HDM2, and SIVA1 expression in the stratum basale of epidermis at day 16 of treatment compared to the control. 

Meanwhile, the proliferation of fibroblast increased with ES with more collagen deposition and faster migration [32,51,52,53,54,55]. ES within physiological strength is not only non-cytotoxic to the fibroblasts [56] but also promotes fibroblast proliferation and migration that aid in wound closure [55]. Moreover, the secretion of FGF-1 and FGF-2 by fibroblasts is significantly upregulated by ES [55], promoting the regulation of cell migration, proliferation, and differentiation [57,58]. As fibroplasia is important for granulation tissue formation, hence, fibroblasts must be able to migrate to cover the wound area. This ability was enhanced by ES in which the directness of fibroblasts was synchronised with the polarisation of integrin α2β1 and lamellipodia formation towards a cathode [59].

Furthermore, ES promotes angiogenesis, whereby endothelial cells migrate and proliferate faster to revascularize the wound [60,61,62]. A previous study with fibroblast and HUVEC illustrated that the ES is beneficial to the vessel formation, since the NOS pathway is activated, upregulating the FGF2 and leading to the cascade activation of the MAPK/ERK pathway, thus promoting the expression of VEGF [61]. Meanwhile, the secretion of FGF-1 and FGF-2 was found to be escalated post-ES on the fibroblasts [55]. It is important to note that FGF-1, FGF-2, and VEGF are angiogenesis factors [63,64], which must be present for effective wound healing. In another study on HUVEC and HMEC, ES increased the migration speed of both cells to the cathode, and the mitosis cleavage plane for both cells was found to be perpendicular to the field vector compared to the random orientation in control on top of the increased production of chemokine receptors CXCR4 and CXCR2 [60], which are crucial for endothelial cell migration [65]. 

### 2.3. Remodelling Phase 

Lastly, ES enhances the remodelling phase by increasing the myofibroblast contractility and collagen conversion from type III to type I on top of the reorganisation of collagen fibres to make scars stronger in terms of tensile strength [54,55]. Rouabhia et al. (2013) demonstrated that by using collagen gel assay, there was a greater contraction of the collagen gel seeded with ES-exposed fibroblast compared to the control with both field strength and exposure duration as the positive factors. Moreover, the expression of α-SMA fibres was only present in the ES-exposed fibroblast but not in the control. This further supports the notion that ES promotes the contractile capacity of fibroblast and trans-differentiation into myofibroblast. In another study using pulsed ES, a similar result was obtained in which ES-activated fibroblast expressed a significantly higher level of α-SMA mRNA in qRT-PCR [54]. A similar finding was found in a study on myofibroblast trans-differentiation whereby ES-exposed HDF expressed a significantly higher level of α-SMA mRNA and TGF-β1 expression with a significant area of contraction in collagen gel contraction assay [66]. Hence, ES promotes the contractile capability of fibroblasts by transdifferentiating into myofibroblasts. 

### 2.4. Other Aspects of Wound Healing Promoted by ES

ES also was found to significantly upregulate the expression of Substance P (neurotransmitter) and Protein Gene Product 9.5 (pan-neuronal marker) on day 14 post-injury at 60- and 30-fold each compared to unwounded skin [67], indicating successful reinnervation. The same study also identified that ES upregulated Class III β-tubulin (TUBB3) and its upstream molecule Factor-Induced Gene 4 with increased TUBB3+ melanocytes, nerve growth factor, and glycoprotein 100, which is a melanocyte-lineage specific antigen indicating the presence of melanogenesis. Hence, ES promotes wound healing with more efficient physiological functions. 

Although lymphatic drainage is not a major step in wound healing, lymphoedema is associated with chronic wounds and impedes wound recovery [68]. Moreover, the lymphatic system is part of the skin organ; therefore, lymphedema pathology must be treated concurrently, especially in the context of the chronic wound [69]. ES has been shown to stimulate lymphatic endothelial cells’ proliferation and migration via the FAK pathway [70]. In a human study, ES increased transient lymphatic activity [71]. The potential therapeutic benefits of ES in the treatment of lymphedema and associated skin ulcers in addition to wound healing stimulation was reviewed and hence strongly recommended [72]. 

Overall, ES promotes all aspects of wound healing. Vasodilatation is enhanced with a faster delivery of more immune cells and elevated phagocytosis in the inflammatory phase. Meanwhile, ES speeds up the transition of a wound into the proliferative phase, which is a keynote to prevent chronic wounds. A more organised re-epithelisation, fibroplasia, and angiogenesis indicate the successful remodelling of a wound into a stronger and scarless wound. Reinnervation, proper pigmentation, and reduction in lymphoedema improve the quality of a healed wound and boost the patient’s confidence upon recovery. These benefits direct our group to the application of ES in wound care. 

## 3. Common Modes of ES

**Figure 4:**
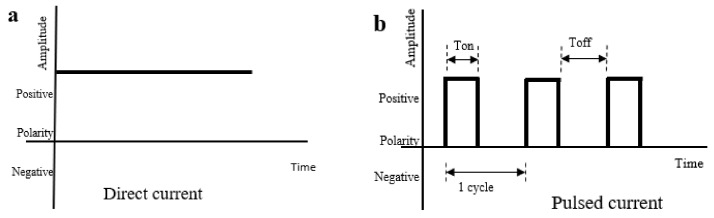

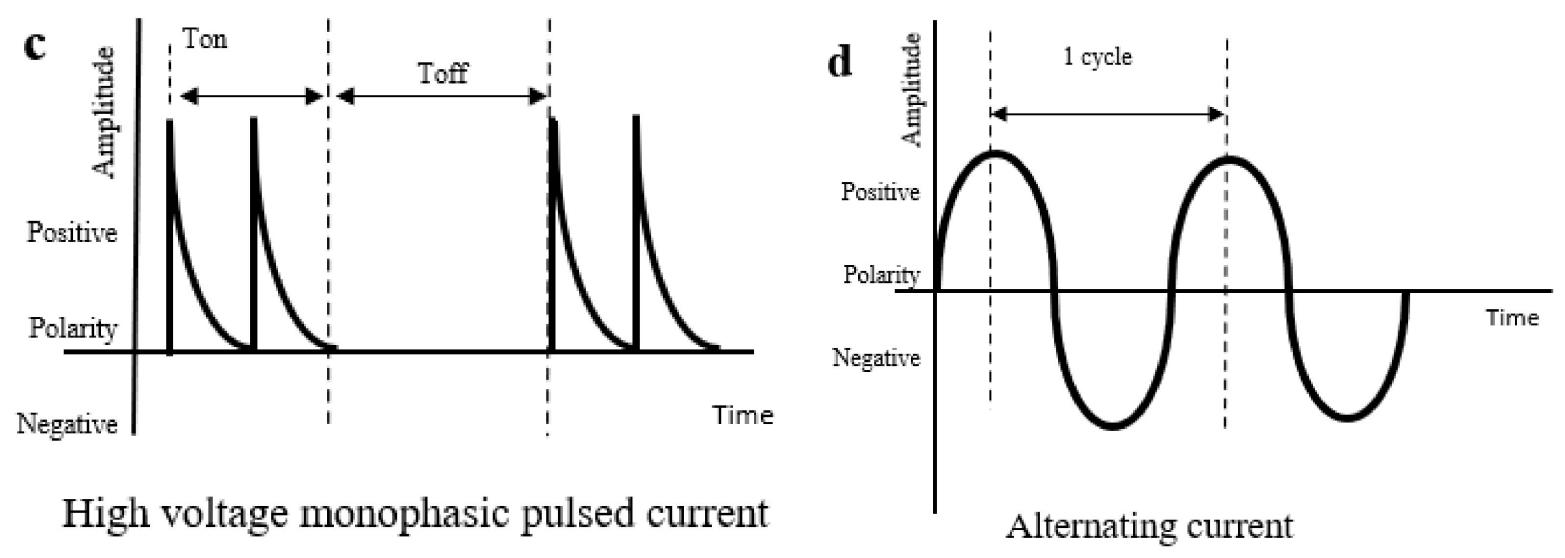
The characteristics of waveform in DC (**a**), PC (**b**), HVMPC (**c**), and AC (**d**) with formulas on basic measurements displayed below. PC with duty cycle of 100% has no pulse and is equivalent to DC.

ES is a term used to describe the application of exogenous electric fields in clinical medicine. ES has been clinically practised since the 19th century when the electrocardiogram (ECG) was introduced to detect heart electrical activity and has improvised to the current 12-lead ECG [73], which is the gold standard investigation to detect myocardial infarction [74]. Concurrently, cardiac pacing was introduced for cardiac resuscitation and technology advances, including cardiac pacemaker and defibrillator [75]. Other than cardiac devices, neuromuscular electrical stimulation (NMES) in sports medicine [76], transcutaneous nerve stimulation (TENS) for chronic pain [77], electroconvulsive therapy in psychiatric disorders [78,79], and deep brain stimulation in Parkinson’s disease [80] are included as ES. There are three common modes of ES, which are direct current (DC), pulsed current (PC), and alternating current (AC). The characteristics of each mode will be described in the next section and shown in Figure 4.

### 3.1. Direct Current (DC)

DC is also known as galvanic current. As shown in Figure 4a, DC is a unidirectional continuous flow of the electric current of the same magnitude in the same direction with the duration of current flow longer than 1 s, giving the DC an absence of waveform [81]. There are two polarities, which are the negative pole named the cathode and the positive pole named the anode. The current flows from the cathode to the anode [82]. In DC, polarity remains fixed unless manually manipulated. A continuous flow of current through an object will produce heat energy known as an electrothermal effect. The excessive electrothermal effect can further cause burn injury to the living tissue and result in an electrophysical effect. Meanwhile, the electrochemical effect can result from a constant application of cathode and anode whereby sodium and chloride ions are attracted, respectively, forming basic sodium hydroxide and acidic hydrochloric acid that lead to chemical burn [83]. The important parameters in the DC ES study are the voltage or current to represent field strength, exposure duration, and polarity.

### 3.2. Pulsed Current (PC)

PC is a uni- or bidirectional pulsing flow of current that lasts shorter than 1 s with a relatively longer inter-pulse interval of no current as shown in Figure 4b [82]. There are many ways to categorise PC depending on the waveform, amplitude, duration, and frequency. PC is widely studied as it has lesser electrothermal, electrophysical, and electrochemical side effects compared to DC due to the pulsing features of PC [48]. Most PC protocols deliver less than 20 mA, and high voltage monophasic pulsed current (HVMPC), as shown in Figure 4c, is the most common PC with twin spikes of high voltage (100–500 V) current delivered in a pair within 2–50 ms [84,85,86,87]. The field strength, exposure duration, and duty cycle affect the galvanotaxis of cells but not the frequency [48]. Hence, the frequency can be adjusted, as PC with a high frequency of 4000 Hz and above is shorter than the nerve refractory period, and the skin senses it with no pain stimulus [52].

### 3.3. Alternating Current (AC)

AC is a continuous bidirectional flow of current that changes its direction and magnitude periodically at least once every second [82]. AC can be delivered in various waveforms that are either symmetrical or asymmetrical but not pulsed. The most common waveform of AC is sine-wave as shown in Figure 4d, in which both phases of the cycles are the mirror images of each other. The polarity of the electrodes in AC changes every cycle, and hence, there is no constant accumulation of heat and chemical by-products over the electrodes in AC. However, AC is not widely studied in wound healing [83].

Period (s): time for a complete cycle including T_on_ and T_off_.
Frequency (Hz): number of complete cycles in a second.
Pulse duration or pulse width (s): time for pulse of current to be on.
Duty cycle (%) = $\frac{T_{\mathrm{on}}}{\left(T_{\mathrm{on}}+T_{\mathrm{off}}\right)}$ × 100

### 3.4. Electrical Studies of In Vitro, In Vivo, and Clinical Trials

ES studies have been conducted in vitro, in vivo, and in clinical trials; these are tabulated in Table 1. In vitro studies mainly show the interaction of ES in wound healing at cellular and molecular levels as reviewed in the wound-healing section above. The galvanotactic response of cells is stronger with increasing field strength, and most cells work effectively within the physiological electric field strength.

Cathodal stimulation is the preferred polarity in wound healing [84,88,89]. The reason for the cathodal preference is explained in Table 2 in which most skin cells migrate to the cathode, although there were studies that stated no difference in wound healing between cathodal or anodal stimulation [85,86]. Oddly, Souza et al. (2018) found that anodal stimulation was better compared to cathodal to stimulate healing and integrate the skin graft [37].

**Table 1:** Key outcomes of various ES studies.

Study Design	Type of ES	ES Field Strength	Exposure Duration	Model	Key Outcome	Reference
In vitro	PC	1, 3, and 5 V	15, 30, and 60 min	HDF	ES increased HDF proliferation with increased expression of PDGFA, FGF2, and TGF-β1.	[52]
In vitro	PC	200 μA Duty cycle: 0%, 10%, 50%, 90%	24 h	HDF	Duty cycle of 10% was viable for HDF and promoted transdifferentiation into myofibroblast.	[66]
In vivo	PC	8 mA	30 min	Rat	Angiogenesis and fibroplasia were promoted.	[90]
In vivo	PC	50 μA	11 days	Rat	Increased fibroplasia, re-epithelisation and angiogenesis.	[91]
RCT	DC	1.48 ± 0.98 mA	An hour daily, 3 d/wk for 4 wk	Type 2 diabetic patients	Reduced wound surface area with improved blood flow to wound.	[88]
Case series	PC	12 ± 1 V	30 min each session, thrice daily, until wound closed	Chronic wounds of various aetiologies	Reduced wound surface area and pain.	[92]
RCT	HVMPC	0.25 A	30 min each session, 4 sessions given	Pressure ulcers	Increased angiogenesis and reduced wound area.	[93]
RCT	HVMPC	subjective dosage (minimum voltage of 100 V)	50 min each session, until detachment of dressing	Burn wounds	ES promoted wound healing and reduced pain in the donor site of the skin graft.	[89]
RCT	PC	20 mA	30 min every other day for 4 weeks	Diabetic foot ulcers	Reduced wound volume and increased blood flow.	[94]

**Table 2:** Galvanotaxis direction of cells and their optimal range of electric field.

Types of Cell	Direction	Optimal Range of Electric Field
Macrophage	Anode	DC/5–300 mV/mm [40]
Monocyte	Cathode	DC/150 mV/mm [40]
HUVEC	Cathode	DC/50–300 mV/mm [60]
HMEC	Cathode	DC/100–300 mV/mm [60]
Keratinocyte cell	Cathode	DC/50–200 mV/mm [95]
Fibroblast cell	Cathode	DC/50–200 mV/mm [55,96,97]

ES has been shown to promote wound healing by shortening the inflammatory phase [98], increasing fibroplasia and collagen deposition [99] with more organised re-epithelisation [53] in addition to angiogenesis [37]. There were studies on diabetic fibroblasts and diabetic rats to simulate wound healing in a diabetic foot ulcer in which the studies showed a promising result of the ES on wound recovery [100,101]. Nicotinic rats were used to mimic arterial ulcers among smokers, and the application of high voltage pulsed current increased the levels of VEGF and FVIII, indicating increased vasodilatation and angiogenesis formation [37]. Although not all models of chronic wounds were studied, the current results provide a good insight into the stimulatory effects of ES on wound care. 

Clinical studies further support the application of ES in wound care. In diabetic foot ulcers, DC is found to increase angiogenesis and vasodilation, leading to increased peri-wound blood flow and reduction of the wound area [88]. The beneficial effect of ES was also seen in other diabetic-related wounds whereby a chronic wound at a post-amputation stump site of nearly 1-year duration closed completely after 45 sessions of ES [102]. In pressure ulcers, amazing recovery was seen in HVMPC [84,85,86,103]. The outcome of ES was comparable to the negative pressure wound therapy (NPWT) in a study of pressure ulcers [104]. 

## 4. ES in Relation to Tissue Engineering and Regenerative Medicine

In recent decades, the three components of tissue engineering—namely, scaffold, cells, and growth factor—have gained popularity in regenerative medicine. The fabrication of an electroconductive scaffold that can generate ES is gaining niches in skin tissue engineering. A new green source of energy empowered from nanogenerators and enzymatic biofuel cells can generate ES to promote wound healing. Stem cell (SC) therapy, which has high differentiation potency, will be reviewed, since ES can control SC proliferation and differentiation to regenerate new skin tissue. As a label-free form of cell manipulation, DEP is a new technology to sort cells and align them in a specific manner, making the wound-healing process organised and potentially could accelerate wound recovery.

### 4.1. Electroconductive Scaffold 

The fabrication of biopolymers such as silk, gelatin, collagen, nanocellulose, chitosan, PVA, and PVP in the wound care context is well-established with high values of biodegradability, renewability, environment-friendliness, non-cytotoxicity, good mechanical strength, low cost, and easy reproducibility [105,106,107]. On the one hand, natural polymers have been proven to be good candidates as skin scaffolds in wound healing since they mimic the components of extracellular matrix [108]. However, they are electrically inert with an absence of free electrons or ions, making them suitable as insulator polymers [109]. There, electroconductive materials play a role to make a scaffold electrically active. Generally, conductive materials can be categorised into organic and inorganic. Organic conductive materials are commonly known as intrinsically conducting polymers (CP), while inorganic conductive materials are mainly carbon- and metal-based materials.

#### 4.1.1. Conducting Polymers (CPs)

CPs were discovered in the 1960s when Hideki Shirakawa worked on polymers from acetylene using the Ziegler–Natta catalyst and accidentally produced a silvery polyacetylene film after making by adding nearly 1000 times the initially planned catalyst concentration. However, the silvery polyacetylene film was not yet a conductor. A collaboration among Shirakawa, Alan Heeger, and Alan MacDiarmid discovered the first electroconductive polymer named polyacetylene, and they were nominated for the Nobel prize in Chemistry 2020 [110]. The conductivity of the polyacetylene film increased by 10 million times within a few minutes at room temperature after being exposed to halogen vapour [111]. Despite the astonishing discovery, polyacetylene was unstable in air and not processable; hence, its further application was halted [112]. Nevertheless, this is an eye-opening finding and has prompted scientists to study other conjugated polymers that have gained attention in biomedical science and tissue engineering [113,114]. 

The common commercialised CPs include polyaniline (PANi), polypyrrole (PPy), and poly(3,4-ethylenedioxythiophene) (PEDOT). The presence of alternating single and double carbon–carbon bonds in CPs render the easy mobilisation of valence electrons, making them polarisable via doping or protonation. However, pure CPs are very stiff due to their rigid backbones, making them very difficult to be tuned [115]. In addition, pure CP application in humans is not practical, as CPs are non-flexible, non-processable, and non-biodegradable with the risk of chronic inflammation in long-term implantation [116]. Nevertheless, the composite of CPs with other biodegradable polymers was shown to be biocompatible [117], providing an opportunity for the fabrication of a biodegradable CP- based scaffold in the field of tissue engineering [118]. The incorporation of other insulating polymers such as silk [119], gelatin [120], chitosan [121,122,123], PLLA [124], polycaprolactone (PCL) [125], and others into CPs can improve the shortcomings.

PANi was first synthesised in 1886 and has become one of the most studied CPs owing to its low cost, easy synthesis, high theoretical specific capacitance, controllable electrical conductivity, and redox properties [126,127]. PANi is the only CP in which the electrical conductivity can be tuned accordingly by the adjusting the doping or protonation [128,129]. A biodegradable film made up of PCL and PANi is shown to be non-cytotoxic and promotes the differentiation of human mesenchymal stem cells into a cardiomyocyte lineage [130]. The same study showed that pure PCL had an electrical conductivity of approximately 3 × 10^−12^ S cm^−1^, and the incorporation of PANi increased the conductivity of the PCL/PANi composite to 8 × 10^−5^ S cm^−1^ when the PANi percentage was 20%. The biocompatibility of PANi was supported by another study on lymphocyte cultures with documented live cell readings of 94.93 ± 1.5%, 94.17 ± 0.76%, and 94.77 ± 1.51% on pure chitosan film, PANi–chitosan 1:10 film, and PANi–chitosan 1:1 film, respectively [122]. Meanwhile, the human-derived cell line ReN-VM was used to access the electrospun PCL–PANi nanofiber, which showed that PCL–PANI (88:12) had a higher growth rate of 0.50 per day compared to 0.45 per day in PCL 13% in addition to the documented electrical conductivity of 7.7 × 10^−2^ S cm^−1^ [125]. Another study showed prolonged and stable electrical conductivity in PANi–chitosan patches despite incubation in the physiological medium phosphate buffer solution for 21 days [123]. 

PPy, another widely studied CP, is the oxidative polymerisation of pyrrole with a formula of H(C_4_H_2_NH)_n_H. As with other CPs, PPy is commonly blended with other biodegradable polymers in tissue engineering. Liang et al. (2021) successfully fabricated conductive PPy-encapsulated silk fibroin (SF) fibres through electrospinning for cardiac tissue engineering, and the team revealed that the ratio of PPy to SF at 15:85 provided sufficient electrical conductivity for cardiomyocytes [131]. By using fibroblasts and OLN-93 neural cells, nanochitosan–PPy film of a 2:10 ratio was shown to be biocompatible and non-cytotoxic with an electrical conductivity of 1 × 10^−3^ S cm^−1^, which is valuable in neural tissue engineering [121]. Three-dimensional (3D)-printable oxidised alginate–gelatin hydrogel incorporated with 0.1 M PPy: PSS was fabricated with greater cell seeding efficiency due to the porous structure created by the 3D printing and 1 × 10^−5^ S cm^−1^ conductivity in dry and 0.015 S cm^−1^ in wet conditions, respectively [120].

PEDOT is a derivative of polythiophenes that has gained attention recently due to its high conductivity, transparency, and stability in the field of conducting polymers with PEDOT:PSS being the most common commercialised form. The fabrication of silk fibroin composite scaffold incorporated with PEDOT:PSS crosslinked with polyvinyl alcohol (PVA) has been shown to have increased electrical conductivity with the increasing concentration of PEDOT:PSS. The 0.1–0.2% PEDOT:PSS is the optimal concentration, whereas 0.3% is cytotoxic to the corneal epithelial cells [119]. Meanwhile, the excellent conductivity of PEDOT was seen in a study of electroconductive hydrogel with iota-carrageenan (CRG), PVA, and PEDOT:PSS with a conductivity of 0.01 S cm^−1^ in both dry gel and distilled water conditions [132]. These findings were supported by another study of poly [2,20-m-(phenylene)-5,50-bibenzimidazole] (PBI) nanofibres coated with PEDOT:PSS via spin and dip-coating methods with a recorded electrical conductivity of 28.3 S m^−1^ and 147 S m^−1^, respectively in addition to bio-compatibility to hBM-MSC [133]. The hydrophilicity of the PLA-PHBV scaffold was also increased by the coating with PEDOT:PSS [134]. Moreover, PEDOT showed stable and prolonged conductivity, since it can retain 78% of its initial current intensity at 100 h in PEDOT-coated poly (L-lactic acid) (PLLA) scaffold, which served as a good conductor for electrical stimulation on HDF [135]. Nevertheless, compositing PEDOT with other biodegradable polymers is crucial to improve bio-compatibility, bio-functionality, cell–material interaction, and reduce inflammation in the biological system [136]. 

#### 4.1.2. Inorganic Conducting Materials

Unlike CPs, inorganic conducting materials are those without carbon–hydrogen bonds. The examples include allotropes of carbon such as graphene, carbon nanotubes, and metallic compounds. Inorganic nanomaterials are on the rise in the platforms of wound healing and tissue engineering due to their outstanding intrinsic properties such as antimicrobial property in silver and silica, antioxidant effect in cerium oxide, reactive oxygen species (ROS) production to promote cell proliferation by zinc oxide, and the electrical conductivity in carbon nanotubes [137]. Next, this paper will review the inorganic conducting materials that can conduct electricity and their niches in skin tissue engineering.

Functional carbon-based nanomaterials such as graphene oxide, carbon nanotubes, and nanodiamond have been explored in the biomedical field due to their excellent electrical conductivity, high mechanical strength, and optical property [138]. Commercially available graphite or diamond nanoparticles were shown to disperse evenly in the PLA matrix with an increase in both AC and DC conductivity of eight orders of magnitudes compared to the pure PLA [139]. Another study showed an even distribution of graphene oxide (GO) nanosheets within methacryloyl-modified decellularised small intestine submucosa hydrogel by coating the GO nanosheets with hydrophilic serum proteins prior to mixing, and the resulted GO-embedded hydrogel was biocompatible and non-cytotoxic to human adipose tissue-derived mesenchymal stem cells [140]. The same study successfully reduced GO in situ by incubation with ascorbic acid at 37 °C for 3 days, and this resulted in a significantly improved electrical conductivity [140]. 

Carbon nanotubes are good conducting materials. Recently, enzymatic biofuel cells emerged as a new fuel source to generate electrical energy through the enzymatic catalysis of biofuels such as sugar [141]. Carbon nanotubes were selected to conduct electricity produced by enzymatic biofuel cells and recorded a voltage of 2.09 V [142]. By adopting the concept of biofuel cells, a bioelectric plaster was fabricated with a current intensity of 1 mA cm^−2^ that lasted for 12 h [143]. An in vivo study of the bioelectric plaster on the full thickness of the rat wound model showed that ES generated a faster wound closure, and the hydrogel provided a moist microenvironment to reduce wound contracture. Unfortunately, this bioelectric plaster is needed to be replaced every 12 h to ensure the continuous generation of electricity. This may cause secondary trauma to newly grown fragile granulation tissues in the wound, although the bioelectric plaster was shown to be adherent but not adhesive. 

MXenes are the 2D transition metal carbides, nitrides, and carbonitrides that are gaining the attention in the field of tissue engineering, biomedicine, energy science, electronic devices, and nanomaterials due to their excellent electrical conductivity, large surface area to volume ratio despite their nanoscale, and strong mechanical strength [144]. The availability of functional groups on the surface of MXenes allows for the surface modification and conjugation with other biocompatible polymers such as proteins and polysaccharides to facilitate its application in the biological system [144]. Interestingly, MXenes have an excellent antibacterial property in which it is stronger compared to GO, and direct contact with MXenes can damage the bacterial cell membranes, giving a bactericidal effect [145]. A study of composite hydrogel made up of bacterial nanocellulose and MXene-2% (rBC/MXene) on a full thickness of rat wound model found that electroconductive MXene significantly increased granulation tissue thickness with a more complete and organised re-epithelisation in addition to angiogenesis and lesser neutrophils infiltration [146]. 

The application of metal elements in wound dressing to generate ES was studied by Yu et al. (2019) with the fabrication of microcurrent dressing using medical cotton cushion with the wound facing one side being sprayed with silver and zinc particles in a dot matrix-arrayed method. This microcurrent dressing was able to generate 0.95 V substantially for 3 days upon being moistened without external electric supply and closed up to 95% of the wound area on day 14 in vivo with a reduced level of inflammatory cytokines of TNF-α and IL-1β in addition to an increased level of growth factors VEGF and EGF [11]. 

### 4.2. Nanogenerators (NG)

A nanogenerator is an electrical energy generator from mechanical energy [147]. Piezoelectric (PENG) and triboelectric (TENG) nanogenerators are examples of nanogenerators [148]. The utilisation of a nanogenerator to produce electricity is another idea other than biofuel cells in the modern ES. 

A wearable NG was made by overlapping the electronegative layer of Cu/PTFE with another electropositive layer of Cu to produce electrical energy when converting the mechanical displacement between the two layers during the breathing movement of the rat [149]. The pulsed ES generated approximately 2.2 V at a rate of 110 per min, which promoted nearly complete wound closure at 72 h in vivo compared to 50% wound closure in the control group. An in vitro study of the nanogenerator using NIH3T3 fibroblasts showed excellent cell viability of 127% and increased proliferation in addition to elevated expression of VEGF, TGF-β, and EGF with a significantly reduced level of ROS.

Another wearable polyacrylamide-based gel containing lithium chloride salts coated with heptadecafluoro-1,1,2,2-tetrahydrodecyl trichlorosilane as TENG was fabricated [150]. This fully stretchable and biocompatible ionic patch was able to generate approximately 2 V when applied on an active rat, and the ES resulted in three times faster wound closure in vivo, whereas an in vitro study on normal and diabetic HDF showed increased proliferation, migration, and expression of FGF, VEGF, and EGF. 

PVDF nanofibres is a piezoelectric material [151]. In another study, electrospun PVDF nanofibers, aluminium foils, and aluminium electrodes were used to make a piezoelectric nanogenerator, which was then attached to the centre of the adhesive hydrogel of polydopamine–polyacrylamide to be applied to the full thickness of the rat wound model. The result showed complete epithelisation on day 10 compared to 22.7% of the wound area remaining open in the control group [152]. The piezoelectric nanogenerator generated an average of 0.1–0.5 V of ES, which leads to an increased expression of CD 31, VEGF-A, and TGF-β1 in the wound with no impact onto the blood, liver, and renal profile.

Nevertheless, the translation of nanogenerator application into human use requires further refinement, as most experiments strip a nanogenerator on the chest of an animal, which is unethical to be applied on a patient. Furthermore, most patients with chronic wounds have comorbidities and are not as physically active as youngsters to generate adequate mechanical energy. Meanwhile, acute extensive burn patients may be haemodynamically unable to perform extensive physical activity in the early wound period, and late treatment can result in contracture. 

### 4.3. Stem Cell (SC) Therapy 

SC therapy is a component in tissue engineering due to its self-renewal ability, and it restores the function of degenerative organs. In skin tissue engineering, SCs harvested from bone marrow, adipose tissue, amniotic fluid, and the umbilical cord can be differentiated into the skin cell lineage and incorporated into the skin scaffold to promote wound healing [153]. By the application of TENG, a pulsed ES of 30 μA was generated at 1.5 Hz that successfully rejuvenated aged bone marrow mesenchymal SC (BMSC) with higher proliferation, pluripotency, and differentiation ability [154]. Meanwhile, ES generated by enzymatic biofuel cells was found to be able to change the cell morphology and gene expression of adipose mesenchymal SC (ASC) [155]. The combination of PC of 0.1 V/cm with a pulse duration of 0.04 ms at 10 Hz for 30 min daily up to 21 days in a 3D nanofibrillar cellulose hydrogel resulted in increased osteogenic potential in ASC [156]. A biphasic PC of 1 V/cm at 0.5 Hz in combination with cyclic strain was found to well differentiate the rat BMSC differentiation into neural cells [157]. ES using AC at 1.7 V and 20 Hz on ASC significantly promoted cell proliferation with a 4.5-fold increase in cell numbers and 2.7-fold increase of cellular surface coverage, and 50.5% of cells entered the proliferative phases of the cell cycle [158]. Hence, ES can assist the proliferation and differentiation of SC into the skin cells of interest and promote wound healing by regenerative medicine.

### 4.4. Dielectrophoresis (DEP)

**Figure 5:**
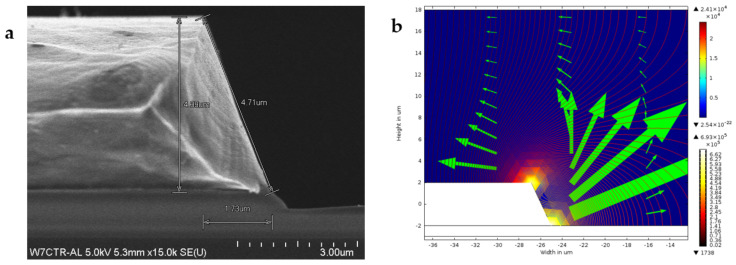
The SEM image of the fabricated microelectrode session of TAMA with measurements is clearly shown in subfigure (**a**). Subfigure (**b**) shows the finite-element method simulation analysis through COMSOL software in which TAMA with a side wall profile of 65 degrees generated the best two spots of high electric field intensity, which subsequently produce lateral and vertical dielectrophoretic forces for particles manipulation. Images are adapted with permission from Buyong et al. (2017).

**Figure 6:**
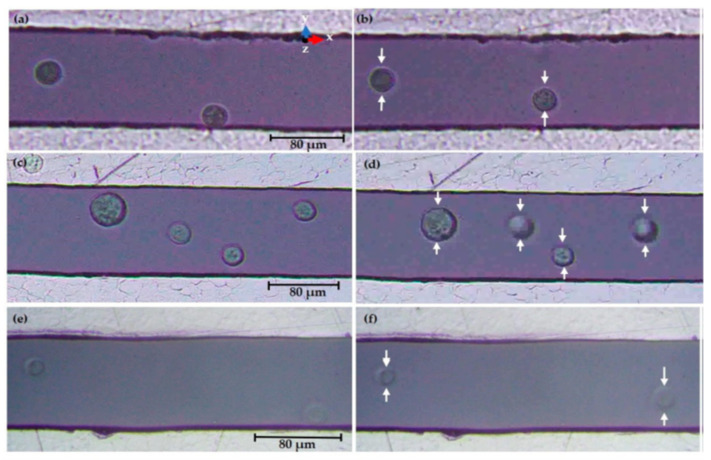
Fluorescence microscopy observations of keratinocytes in DMEM/F-12 conductive medium showed that the keratinocytes experienced dielectrophorectic force and migrated to the area of lowest electric field strength, which was the middle between electrodes at a final 10 VPP at 300 kHz (**b**), 800 kHz (**d**), and 15 MHz (**f**) compared to the initial 0 VPP at 300 kHz (**a**), 800 kHz (**c**), and 15 MHz (**e**). Image is adapted with permission from Deivasigamani et al. (2021).

AC is not commonly applied in wound healing, which is possibly due to the lack of polarity for galvanotaxis. Nevertheless, dielectrophoresis (DEP) is a form of electrokinetic phenomenon requiring AC to generate a non-uniform electric field in order to manipulate the movement of neutral particles [159]. DEP has been well known to be cheap, fast, and label-free with high sensitivity and specificity for cell sorting and particle separation [160,161]. The dielectrophoretic force can be manipulated by adjusting the input frequency of alternating current and conducting medium while the geometry and dielectric properties of the cells are not fixed [162,163]. However, the interaction of DEP in mobilising a particular keratinocyte or fibroblast and aligning them in a proper plane remains to be elucidated. The fabrication of tapered aluminium microelectrode arrays (TAMA) started with a silicon substrate as the base of the microelectrode, which was then topped with silicon oxide followed by titanium/titanium nitrite and then aluminium/silicon/copper composite with photoresist and lastly the etching process [161]. Figure 5a shows the scanning electron microscope (SEM) image of the fabricated TAMA electrode. TAMA work led to the identification of a tapered side wall between 60 and 70 degrees for the generation of a more uniform electric field from two spots at top and bottom edges of the tapered microelectrodes [161]. Refinement of the TAMA work concluded that 65 degrees was the best reading in TAMA to generate dielectrophoretic force for lateral and vertical manipulation of particle manipulation as shown in Figure 5b [164]. A DEP study on keratinocytes using “MyDEP” software showed that the DEP response of keratinocytes was successfully simulated by using polystyrene particles [164]. This is of interest, as DEP targeted specific cells and hence, the mobilisation of keratinocytes in an appropriate direction, and the plane will make re-epithelisation possible, especially in the case of a chronic wound with sufficient granulation but slow or no opposing wound edges. Figure 6 shows successful manipulation of DEP on keratinocytes into area of interest Nevertheless, more studies of DEP in wound healing need to be explored. 

## 5. Conclusions

Wound care is a world burden, while clinically practised skin grafts produce a risk of graft failure, donor site infection, and other complications. The abilities of ES to restore the weakened endogenous current of injury in chronic wounds offer an astounding opportunity to expedite wound recovery. ES shortens the inflammatory phase, and it also enhances fibroplasia, re-epithelisation, and angiogenesis together with remodelling to form a stronger scar in well-established in wound care. 

HVMPC has overall good outcomes in chronic wounds; however, the presence of electrothermal, electrophysical, and electrochemical side effects, although lesser than the DC, must be taken into clinical consideration. Meanwhile, nanogenerators, a green form of electric energy converted from mechanical energy, require an amount of physical movement, which can be difficult in patients with comorbidities in addition to discomfort brought by stripping a nanogenerator to the human chest. 

Undeniably, electroconductive materials are interesting to be incorporated as part of scaffolds to assist wound healing. However, pure conducting materials are not easily processable or biodegradable with a potential risk of inducing inflammation. Hence, the incorporation of conducting materials into biodegradable polymers of both natural and synthetic origin is widely practised and highly encouraged. Further fine-tuning of electroconductive scaffolds mimicking the targeted extracellular matrix shall direct researchers to a biodegradable, biocompatible, non-cytotoxic, non-immunogenic, easily synthesised, and low-cost fabrication for popular application. 

Bioelectric plaster-like wound dressings with electroconductive materials or enzymatic biofuel cells are examples of smart wound dressings without an external power supply. However, the frequent changing of wound dressing should be avoided to prevent unnecessary pain and secondary trauma to the fragile growing granulation tissue. 

Essentially, a successful wound closure within a short period in a painless procedure is the ultimate fundamental of research on wound healing. With the understanding of the current of injury, ES is believed to accelerate wound healing and replace skin grafting in the future either through electric devices or smart wound dressings. 

## 6. Future Direction

DEP is a label-free cell patterning that mobilises specific cells according to the dielectrophoretic force exerted under a non-uniform electric field. The potential ability of DEP to mobilise keratinocytes and fibroblasts specifically into the top and bottom layers mimicking the structure of epidermis and dermis is a field of interest in wound care. In addition, DEP applies alternating current to run through electrodes, and hence, the accumulation of heat and chemicals at a particular electrode can be avoided. Nevertheless, the interaction of ES on wound microbial propagation remains to be elucidated. 

## Data Availability

No new data were created or analyzed in this study. Data sharing is not applicable to this article.
